# Supplementary material for: Preeclampsia promotes autism in offspring via maternal inflammation and fetal NFκB signaling
Source: Life Sci Alliance. 2023 Jun 8;6(8):e202301957. doi: 10.26508/lsa.202301957 (PMC10250690; doi:10.26508/lsa.202301957)
Supplement: Supplementary file 7 [file LSA-2023-01957_Supplemental_Data_1.docx]

**Supplementary Materials and Methods**

**Preeclampsia promotes autism in offspring via maternal inflammation and fetal NFκB signaling**

**Blood pressure measurement and water consumption**

Due to the presence of dark skin in C57BL/6J mice, we cleaned their tails with 100% ethanol prior to experiments. For pilot experiments, we used untreated pregnancy C57BL/6J female mice with matched ages and CD-1 mice as a reference to establish appropriate parameters for blood pressure measurement using a tail-cuff monitor (BP2000 Analysis System; Visitech Systems, Apex, NC) on C57BL/6J pregnant mice (1). Additionally, the water intake of each mouse per group was measured from pregnancy day 10 through day 18, following the instruction from a previous report (2).

**ASD behavioral tests**

Open filed test (OFT) was used to assess the motor function and anxiety level of each mouse. Each mouse was placed in a box (40 cm wide x 40 cm long x 40 cm high) for free moving. The bottom surface of the box was divided into 9 parts of equal size with computer software. The center part was defined as the center zone. The positions and trajectories of the mice over a 20-min period were recoded and analyzed by the software. The total distance travelled and the time spent in the center zone were indicated the locomotor activity and anxiety level of mouse.

Elevated plus maze (EPM) was used to evaluate the anxiety level of each mouse. The apparatus in EPM is a cross with 2 open arms (30 cm x 6 cm) and 2 closed arms (30 cm x 6 cm), which was elevated 45 cm above the floor. Each mouse was placed in the center of the cross, facing one of the closed arms and its behavior was recoded for 5 min. The time spent in the open arms and closed arms was used to assess anxiety level.

The marble burying test was used to assess the repetitive behaviors of the mice. In the habituation trial, the mice were individually placed in testing cages (30 cm x18 cm x 15 cm) filled with corn bedding material to a depth of 3-4 cm. At the end of a 20- min habitation period, the mice were gently removed from the testing cages, and 20 glass marbles were laid out in four rows of five marbles at 3-cm intervals on the surface of the bedding material. Then, the mice were returned to the testing cages for free exploring. After that, marbles covered ＞50% by bedding were counted manually (3).

Three-chambers test was used to assess the social behavior of the mice. An opaque plastic box (20 cm x 15 cm x 25 cm) partitioned by transparent plastic walls with an opening (10 cm) in the center, was used in the experiment. The test was divided into 3 sessions. In the habituation session, the experimental mice were placed in the center chamber and explored for 20 min freely. In the test session, the experimental mice were permitted to explore the left and right chambers. The left chamber contained an unfamiliar C57BL/6 mouse of matched sex, age, and size, which was placed in a small wire cylinder (8 cm in diameter and 15.5 cm in height) and served as a social stimulus. The right chamber contained an identical empty cylinder and served as a nonsocial stimulus. During10-min exploration period, approach behavior, such as sniffing, and approaching of the targets in each chamber, was recorded and analyzed. A sociability quotient (social-nonsocial/total time in middle chamber) was also calculated.

**Morris Water Maze**

Sixteen adult offspring per gender from each group were subjected to the Morris Water Maze test to evaluate spatial learning and memory. The protocol was modified based on previous references (4, 5). Briefly, the test was divided into two sessions: training and probe sessions. During the training session, the mice were placed in a white polyethylene tank with a diameter of 120 cm and filled with water to a height of 34 cm. Non-toxic white Crayola paint was added to the water to make it opaque, and the water temperature was maintained at 24.0±0.5℃. A hidden platform measuring 10×10 cm was placed below the water surface. The testing room was decorated with spatial cues to help the mice remember the platform's location. A camera was mounted above the center of the tank to record the mouse's behavior using a video tracking system (Noldus Information Technology, Wageningen, The Netherlands). The mice were allowed to swim to the platform and escape the water within 60 seconds. If the time limit was exceeded, the mice were gently directed to the platform, which was located in the target quadrant (Northwestern, NW). The mice were allowed to sit on the platform for 10 seconds before being returned to their holding cages after successfully climbing onto the platform. A total of 5 training trials were conducted. Following the training session, the probe session was conducted on the next day. During this trial, the platform was removed, and the time spent in each of the four quadrants by the mice was recorded within 60 seconds.

**Calcium phosphate transfection**

Conditioned medium from the cultured neurons was obtained the day before transfection and stored in a refrigerator. In a 1.5 ml Eppendorf tube, 2 μg DNA vector was added to 100 μl CaCl_2_ solution. Then, the DNA-CaCl_2_ mixture was slowly added to 100 μl BBS solution, while flicking the tube. The new mixture was kept away from light for 20 min at room temperature, and then the DNA-CaCl_2_-BBS mixture was added to each well to cover the cells. After 90 min of incubation at 37℃ in 5% CO_2_, the cells were rinsed with wash buffer (pH7.4, 1 mM CaCl_2_, 0.8 mM MgSO_4_, 5.3 mM KCl, 120 mM NaCl, 10 mM HEPES and 25 mM glucose) and cultured in 0.5 ml conditioned media.

**Golgi staining**

Mouse Brains were quickly removed and immersed in Golgi staining buffer (solution A/B=1:1) for 2 weeks in the dark. Next, the tissues were transferred into Solution C in the kit and stored at room temperature in the dark for at least 72 hours. Finally, the brains were sliced at 100 μm thickness, and the stained slices were plated on gelatin-coated glass slides and mounted using neural resin. To exclude the effects of the behavioral tests, littermate mice that were not subjected to the behavior test, were used in this assay. At least 3 mice of each sex from 2 different litters and at least 6 slices from each brain were used in the experiment. The stained cells in cortex and hippocampus were imaged with a confocal microscopy under a 20x objective. And the basal dendrites of layer2/3 pyramidal neurons and CA1-CA3 hippocampal neurons were imaged at 1-μm Z intervals with a confocal microscope (Leica Microsystems, Wetzlar, Germany), using a 63x oil immersion objective (N.A.=1.4) and 2x optical zoom. The number of spines on dendrite longer 20 μm was counted using ImageJ software (NIH, USA).

**Haematoxylin and eosin staining**

The slices were immersed in haematoxylin for 1 min and eosin for 10 s. Excess dye was rinsed with ddH_2_O. Images were acquired under a microscope. The distance from cerebral ventricles to the meninges was measured using Image Pro Plus software and used as a measure of the thickness of the cortex.

**Immunofluorescence (IF)**

The embryos and pups were perfused with 5ml of PBS first, followed by 5 ml 4% PFA via heart perfusion to achieve a good fixation result. The mouse brains were post-fixed in 4% PFA overnight and equilibrated in 30% sucrose/PBS. The tissues were embedded in OCT and coronally sectioned at 10 μm. For BrdU staining, the slices were pretreated with antigen retrieval buffer at 96℃for 20 min and slowly cooled to room temperature. Next, the slices were incubated in 2N HCl at 37℃ for 13 min, and washing with Na_2_Ba_4_O_7_ (0.1 M, pH 8.5) for 5 min and PBS 3 times for 15 min.

All fluorescence images were obtained at 1-μm interval in z-axis with an SP8 confocal microscope. The number of signaling cells in sections, total dendrite length from soma to the terminal and soma size of neuroprogenitors were analyzed with Image Pro Plus and ImageJ, respectively.

**Western blots**

After protein extraction, the samples were loaded on 10% SDS-polyacrylamide gels and processed according to standard Western blotting protocols. The intensity of each band was quantified using ImageJ software (NIH).

**Statistics**

Statistical analyses were performed using Graphpad Prism or SPSS software. Normality and equal variances between groups were assessed using Kolmogorov-Smirnov test. Student’s test, multiple t tests with Holm-Sidak correction, or One way ANOVAs and Two-ways ANOVA with post hoc Tukey correction for multiple comparisons test were performed when normality and equal variance were achieved between groups. If not, Kruskal-Wallis test with Dunn’s correction or Mann-Whitney U-test or Wilcoxon signed-rank test were carried out, respectively. All data are represented as mean±s.e.m. Sample sizes were determined by previous protocols for each assay, without any statistical methods. Data were not preprocessed except that RT-qPCR and western blot data were normalized to the housekeeping genes, and the diameters of neurospheres in the cumulative distribution assay. For experiments that were carried out at different developmental stages, statistical analyses were determined based on the control group at each specific time point, and comparisons among different developmental stages were not analyzed. For all behavioral assays, at least 4 litters/group were used and variability within and between litters were assessed by univariate analysis of general linear model and Levene’s test, for homogeneity of variance, exhibiting similarity across test groups which could exclude the litter effect. The statistical significance were set at p＜0.05. The detailed statistic values were following:

**Fig.1:**

Panel A, brain weight: multiple t test with Holm-Sidak correction.

control E15.5 vs PE E15.5, p=0.814, Variance Levene test, F=3.506, p=0.12;

control E17.5 vs PE E17.5, p=0.002, Variance Levene test, F=3.063, p=0.163;

control P0 vs PE P0, p=0.05, Variance Levene test, F=1.891, p=0.42.

Panel C, cortex thickness: multiple t test with Holm-Sidak correction for each time-point.

control E15.5 vs PE E15.5, region 1, p＜0.001, Variance Levene test, F=2.12, p=0.553. region 2, p＜0.001, Variance Levene test, F=7.831, p=0.125; region 3, p=0.755, Variance Levene test, F=1.231, p=0.868; region 4, p=0.781, Variance Levene test, F=1.111, p=0.933;

control E17.5 vs PE E17.5: region 1, p＜0.001; Variance Levene test, F=1.493, p=0.75; region 2, p＜0.001; Variance Levene test, F=2.785, p=0.423. region 3, p＜0.001; F=3.931, p=0.291; region 4, p＜0.001, Variance Levene test, F=9.493, p=0.097;

control P0 vs PE P0: region 1: p＜0.001, Variance Levene test, F=2.11,p=0.555; region 2, p＜0.001, Variance Levene test, F=1.56, p=0.724; region 3, p＜0.001, Variance Levene test, F=2.561, p=0.46; region 4, p=0.002, Variance Levene test, F=2.19, p=0.536.

Panel E, 4h BrdU incorporation assay, multiple t test with Holm-Sidak correction.

Control E15.5 vs LO E15.5, p=0.168; Variance Levene test, F=0.1.682, p=0.68;

Control E17.5 vs LO E17.5, p=0.008; Variance Levene test, F=1.631, p=0.68;

Control P0 vs LO P0, p＜0.001; Variance Levene test, F=5.556, p=0.193.

Panel F, PH staining assay, multiple t test with Holm-Sidak correction.

Control E15.5 vs LO E15.5, p=0.936; Variance Levene test, F=1.005, p=0.997;

Control E17.5 vs LO E17.5, p=0.025; Levene test, F=1.403, p=0.788;

Control P0 vs LO P0, p=0.006; Variance Levene test, F=1.387, p=0.795.

**Fig.2:**

Panel B:

multiple t test with Holm-Sidak correction;

Control E15.5 vs PE E15.5, p=0.777;

Control E17.5 vs PE E17.5, p＜0.001;

Control P0 vs PE P0, p＜0.001.

Panel C:

non-parametric Kolomogorov-Smirnov test.

Control E15.5 vs PE E15.5, p=0.002;

Control E17.5 vs PE E17.5, p＜0.001;

Control P0 vs PE P0, p＜0.001.

Panel G:

Multiple t test with Holm-Sidak correction

*Ube2c*: p=0.012

*Celsr1*: p=0.04

*Kif18*: p=0.047

*Creb5*: p=0.019

*Gli2*: p=0.048

*Plk1*: p=0.003

**Fig.3:**

Panel A:

Multiple t test with Holm-Sidak correction, body weight offspring Control vs offspring, P0, p＜0.001; P2, p＜0.001; P3, p＜0.001; P6, p=0.911; P9, p=0.808; P12, p=0.41; P15, p=0.989; P18, p=0.186; P21, p=0.832; P28, p=0.979; P35, p=0.371; P42, p=0.14; P49, p=0.765; P56, p=0.292.

Panel C:

for male mice, Unpaired Student’s test, p＜0.001, Variance Levene test, F=13.709, p=0.001;

for female mice, unpaired Student’s test, p＜0.001, Variance Levene test, F=35.028, p＜0.001.

Panel D:

for male mice, unpaired Student’s test, p＜0.001, Variance Levene test, F=1.058, p=0.31;

for female mice, unpaired Student’s test, p＜0.001, Variance Levene test, F=7.916, p=0.008.

Panel E:

for male mice, unpaired Student’s test, p=0.003, Variance Levene test, F=2.456, p=0.125;

for female mice, unpaired Student’s test, p＜0.001, Variance Levene test, F=1.16, p=0.288.

Panel F:

for male mice, One-way ANOVA, Tukey HSD, F(3, 76)=0.001.

Control (empty) vs Control (stranger), p=0.003;

PE (empty) vs PE (stranger), p=0.945;

Control (empty) vs PE (empty), p=0.998;

Control (empty) vs PE (Stranger), p=0.984;

Control (Stranger) vs PE (Stranger), p=0.01;

Control (Stranger) vs PE (empty), p=0.002;

for female mice, One-way ANOVA, Tukey HSD, F(3, 76)＜0.001.

Control (empty) vs Control (stranger), p＜0.001;

PE (empty) vs PE (stranger), p＜0.001;

Control (empty) vs PE (empty), p=0.369;

Control (empty) vs PE (Stranger), p＜0.001;

Control (Stranger) vs PE (Stranger), p=0.997;

Control (Stranger) vs PE (empty), p＜0.001.

Panel H:

Multiple t test with Holm-Sidak correction, adult offspring hippocampus control vs PE

*Mnt*: p=0.005;

*Rxra*: p=0.009;

*Ube3a*: p=0.013;

*Arid1b*: p=0.034;

*Cort*: p=0.016.

**Fig.4:**

Panel A:

Multiple t test with Holm-Sidak correction, Control serum vs PE serum, IL4, p=0.88; TNFα, p=0.032; IL17, p=0.83; IL2, p=0.806; IL28, p=0.639; IFNγ, p=0.361; MIP3a, p=0.503; IL1b, p=0.77; IL12p70, p=0.449; IL5, p=0.933; IL6, p=0.842; IL10, p=0.599; IL17F, p=0.516; IL23, p=0.569; IL13, p=0.833; IL21=0.681; TGFβ1, p=0.227; IL22, p=0.323.

Control fetal cortex vs PE fetal cortex, IL4, p=0.518; TNFα, p=0.772; IL17, p=0.2; IL2, p=0.654; IL28, p=0.420; IFNγ, p=0.231; MIP3a, p=0.135; IL1b, p=0.662; IL12p70, p=0.067; IL5, p=0.178; IL6, p=0.088; IL10, p=0.274; IL17F, p=0.05; IL23, p=0.89; IL13, p=0.589; IL21=0.815; TGFβ1, p=0.559; IL22, p=0.805.

Total serum proteins in control and PE, unpaired Student’s test, p=0.456. Variance Levene test, F=1.654, p=0.594.

Panel B:

unpaired Student’s test, placenta control E17.5 vs PE E17.5, p＜0.001, Variance Levene test, F=11.449, p=0.002;

Panel D:

Multiple t test with Holm-Sidak correction, control cortex E17.5 vs PE cortex E17.5, S468, p＜0.001, S539, p＜0.001, total NFκB, p=0.109.

**Fig.5:**

Panel B:

One-way ANOVA, Tukey HSD, F(3,14)=14.845, p＜0.001;

Control vs L-NAME: p=0.752;

Control vs TNFα: p＜0.001;

Control vs TNFα+Bay: p=1;

L-NAME vs TNFα: p=0.001;

L-NAME vs TNFα+Bay: p=0.769;

TNFα vs TNFα+Bay: p＜0.001.

Panel C:

non-parametric Kolomogorov-Smirnov test;

Control vs L-NAME: p=0.583;

Control vs TNFα: p＜0.001;

Control vs TNFα+Bay: p=0.141;

L-NAME vs TNFα: p＜0.001;

L-NAME vs TNFα+Bay: p=0.052;

TNFα vs TNFα+Bay: p＜0.001.

Panel E:

One-way ANOVA, Tukey HSD; F(3,152)=14.709, p＜0.001;

Control vs L-NAME: p=0.666; Control vs TNFα: p＜0.001;

Control vs TNFα+Bay: p=0.79;

L-NAME vs TNFα: p＜0.001; L-NAME vs TNFα+Bay: p=0.995; TNFα vs TNFα+Bay: p＜0.001.

Panel F:

One-way ANOVA, Tukey HSD; F (3,152)=11.255, p＜0.001;

Control vs L-NAME: p=0.796;

Control vs TNFα: p＜0.001;

Control vs TNFα+Bay: p=0.481;

L-NAME vs TNFα: p＜0.001;

L-NAME vs TNFα+Bay: p=0.961;

TNFα vs TNFα+Bay: p＜0.001.

Panel H:

One-way ANOVA, Tukey HSD; F(3,94)=5.017, p=0.003;

Control vs L-NAME: p=0.762;

Control vs TNFα: p=0.002;

Control vs TNFα+Bay: p=0.63;

L-NAME vs TNFα: p=0.037;

L-NAME vs TNFα+Bay: p=0.998;

TNFα vs TNFα+Bay: p=0.041.

Panel J:

Two-way ANOVA, Tukey's multiple comparisons test;

p-S468 Nfκb:

Control NPC vs TNFα-NPC, p＜0.001;

L-NAME-NPC vs TNFα-NPC, p＜0.001;

TNFα-NPC vs TNFα+Bay-NPC, p＜0.001;

other comparisons, p＞0.05;

p-S536, Nfκb:

Control NPC vs TNFα-NPC, p＜0.001;

L-NAME-NPC vs TNFα-NPC, p＜0.001,

TNFα-NPC vs TNFα+Bay-NPC, p＜0.001;

L-NAME-NPC vs TNFα-NPC, p＜0.001,

TNFα-NPC vs TNFα+Bay-NPC, p＜0.001;

Control-Neuron vs TNFα-Neuron, p=0.034;

L-NAME-Neuron vs TNFα-Neuron, p=0.039;

TNFα-Neuron vs TNFα+Bay-Neuron, p=0.013

other comparisons, p＞0.05;

total Nfκb:

all comparisons, p＞0.05.

**Fig.6:**

Panel B:

for male offspring, One-way ANOVA, Tukey HSD, F(3,76)=26.025, p＜0.001; Control+IgG vs Control+antibody, p=0.877; Control+IgG vs PE+IgG, p＜0.001; Control+IgG vs PE+antibody, p=1; Control+antibody vs PE+IgG, p＜0.001; Control+antibody vs PE+antibody, p=0.902; PE+IgG vs PE+antibody, p＜0.001;

for female offspring, One-way ANOVA, Tukey HSD, F(3,80)=13.56, p＜0.001; Control+IgG vs Control+antibody, p=0.27; Control+IgG vs PE+IgG, p＜0.001; Control+IgG vs PE+antibody, p=0.759; Control+antibody vs PE+IgG, p＜0.001; Control+antibody vs PE+antibody, p=0.812; PE+IgG vs PE+antibody, p＜0.001.

Panel C:

for male offspring, One-way ANOVA, Tukey HSD, F(3,72)=4.185, p=0.009; Control+IgG vs Control+antibody, p=0.989; Control+IgG vs PE+IgG, p=0.039; Control+IgG vs PE+antibody, p=0.944; Control+antibody vs PE+IgG, p=0.119; Control+antibody vs PE+antibody, p=0.833; PE+IgG vs PE+antibody, p=0.008;

for female offspring, One-way ANOVA, Tukey HSD, F(3,68)=7.181, p＜0.001; Control+IgG vs Control+antibody, p=0.427; Control+IgG vs PE+IgG, p=0.017; Control+IgG vs PE+antibody, p=0.996; Control+antibody vs PE+IgG, p＜0.001; Control+antibody vs PE+antibody, p=0.604; PE+IgG vs PE+antibody, p=0.015.

Panel D:

for male offspring, One-way ANOVA, Tukey HSD, F(3,74)=10.206, p＜0.001; Control+IgG vs Control+antibody, p=0.422; Control+IgG vs PE+IgG, p＜0.001; Control+IgG vs PE+antibody, p=0.66; Control+antibody vs PE+IgG, p＜0.001; Control+antibody vs PE+antibody, p=0.975; PE+IgG vs PE+antibody, p＜0.001;

for female offspring, One-way ANOVA, Tukey HSD, F(3,64)=23.264, p＜0.001; Control+IgG vs Control+antibody, p=0.975; Control+IgG vs PE+IgG, p＜0.001; Control+IgG vs PE+antibody, p=0.987; Control+antibody vs PE+IgG, p＜0.001; Control+antibody vs PE+antibody, p=0.987; PE+IgG vs PE+antibody, p＜0.001.

Panel E:

for male offspring, One-way ANOVA, Tukey HSD, F(7, 120)=7.439, p＜0.001; Control+IgG (empty) vs Control+IgG (stranger), p=0.008; Control+antibody(empty) vs Control+antibody(stranger), p=0.001; PE+IgG(empty) vs PE+IgG(stranger), p=0.997; PE+antibody(empty) vs PE+antibody(stranger), p=0.047.

Panel F:

for female offspring, One-way ANOVA, Tukey HSD, F(7, 120)=6.448, p＜0.001; Control+IgG (empty) vs Control+IgG (stranger), p=0.026; Control+antibody(empty) vs Control+antibody(stranger), p=0.021; PE+IgG(empty) vs PE+IgG(stranger), p=0.02; PE+antibody(empty) vs PE+antibody(stranger), p=0.047.

**Fig. S1:**

Panel B:

MAP, multiple t test with Holm-Sidak correction, control vs PE, E10, p=0.258; E11, p=1.0; E12, p=0.62; E13, p＜0.001; E14, p＜0.001; E15, p＜0.001; E16, p＜0.001; E17, p＜0.001; E18, p＜0.001.

Panel C:

proteinurine, control vs PE, unpaired Student’s test, p=0.001. Variance Levene test, F=9.261, p=0.012.

Panel D:

Water consumption, multiple t test with Holm-Sidak correction; All comparison, p＞0.05

Panel F:

body weight, multiple t test with Holm-Sidak correction,

control E15.5 vs PE E15.5, p=0.508, Variance Levene test, F=2.292, p=0.296;

control E17.5 vs PE E17.5, p=0.023, Variance Leven test, F=2.811, p=0.196;

control P0 vs PE P0, p＜0.001, Variance Levene test, F=4.667, p=0.06.

Panel G:

mortality, Kolomogorov-Smirnov normality test, control: p＜0.001, PE: p=0.018; Mann-Whitney U-test, control vs PE, p＜0.001.

Panel H:

litter number, control vs PE, Unpaired Student’s test, p=0.853, Variance Levene test, F=0.569, p=0.457.

Panel I:

delivery day, control vs PE, Wilcoxon signed-rank test, p＞1.

**Fig.S2:**

Panel B, brain/body weight ratio: Multiple t test with Holm-Sidak correction, control E15.5 vs PE E15.5, p=0.452, Variance Levene test, F=1.69, p=0.505;

control E17.5 vs PE E17.5, p=0.064, Variance Levene test, F=1.38, p=0.682;

control P0 vs PE P0, p=0.006, Variance Levene test, F=1.826, p=0.445.

Panel C, striatum size: Multiple t test with Holm-Sidak correction,

control E17.5 vs PE E17.5, p=0.469, Variance Levene test, F=1.67, p=0.515;

control P0 vs PE P0, p=0.015, Variance Levene test, F=1.038, p=0.962.

Panel E, Nestin^+^ cell ratio, Unpaired Student’s test, p=0.496, Variance Levene test, F=1.517, p=0.696.

Panel G, adult brain weight, One-way ANOVA, Tukey HSD; F(3, 28)=1.367; p=0.2733;

Control-male vs PE-male, p=0.907;

Control-female vs PE-female, p=0.284;

Control-male vs Control-female, p=0.831;

Control-male vs PE-female, p=0.764;

Control-female vs PE-male: p=0.998;

PE-male vs PE-female: p=0.369.

**Fig.S3:**

Panel A, E17.5 cortex control PE, unpaired Student’s test, p=0.017. Variance Levene test, F=0.9, p=0.365.

Panel B, E17.5 cortex control vs PE, Multiple t test with Holm-Sidak correction, *Grin2a*, p=0.046, *Grin2b*, p=0.005;

Panel C, adult hippocampus control vs PE, Multiple t test with Holm-Sidak correction, *Arid1b*, p＜0.001; *Mnt*, p＜0.001.

**Fig.S4:**

Panel A, for male offspring, unpaired Student’s test, p=0.029. Variance Levene test, F=1.551, p=0.347; for female offspring, unpaired Student’s test, p=0.013. Variance Leven test, F=1.282, p=0.594.

Panel B, for male offspring, unpaired Student’s test, p=0.002. Variance Levene test, F=5.491, p＜0.001; for female offspring, unpaired Student’s test, p=0.805. Variance Levene test, F=7.991, p＜0.001.

Panel C, for male offspring, unpaired Student’s test, p=0.882. Variance Levene test, F=1.065, p=0.8927; for female offspring, unpaired Student’s test, p=0.966. Variance Levene test, F=1.187, p=0.712.

Panel D, for male offspring, multiple t test with Holm-Sidak correction for each trail. Trail 1: p=0.07; Trail 2: p=0.034; Trail 3: p＜0.001；Trail 4: p=0.007; Trail 5: p＜0.001.

for female offspring, multiple t test with Holm-Sidak correction for each trail. Trail 1: p=0.11; Trail 2: p=0.015; Trail 3: p=0.56；Trail 4: p=0.103; Trail 5: p＜0.001.

Panel E, for male offspring, multiple t test with Holm-Sidak correction for each quadrant. NW: p＜0.001; NE: p＜0.001; SE: p＜0.01；SW: p=0.003.

for female offspring, multiple t test with Holm-Sidak correction for each quadrant. NW: p＜0.001; NE: p=0.526; SE: p=0.169；SW: p=0.004.

**Fig.S6:**

Panel B: One-way ANOVA, Tukey HSD; F(3, 586)=172.82;

Control-male vs PE-male, p＜0.001;

Control-female vs PE-female, p＜0.001;

Control-male vs Control-female, p=1;

Control-male vs PE-female, p＜0.001;

Control-female vs PE-male: p＜0.001;

PE-male vs PE-female: p=0.934.

Panel D: One-way ANOVA, Tukey HSD; F(3,593)=41.664, p＜0.001;

Control-male vs PE-male, p＜0.001;

Control-female vs PE-female, p＜0.001;

Control-male vs Control-female, p=0.774;

Control-male vs PE-female, p＜0.001;

Control-female vs PE-male: p＜0.001;

PE-male vs PE-female: p=0.980.

**Fig.S7:**

Panel B: One-way ANOVA, Tukey HSD, F(3,116)=2.132, p=0.1;

Control vs L-NAME: p=0.984;

Control vs TNFα: p=0.99;

Control vs TNFα+Bay: p=0.155;

L-NAME vs TNFα: p=0.968;

L-NAME vs TNFα+Bay: p=0.299;

TNFα vs TNFα+Bay: p=0126.

**Fig.S8:**

Panel A: for male offspring, One-way ANOVA, Tukey HSD, F(3,72)=9.172, p＜0.001;

Control+IgG vs Control+antibody, p=0.74; Control+IgG vs PE+IgG, p＜0.001; Control+IgG vs PE+antibody, p=0.617; Control+antibody vs PE+IgG, p=0.003; Control+antibody vs PE+antibody, p=1; PE+IgG vs PE+antibody, p=0.002;

for female offspring, One-way ANOVA, Tukey HSD, F(3,72)=6.387, p＜0.001;

Control+IgG vs Control+antibody, p=0.876; Control+IgG vs PE+IgG, p=0.07; Control+IgG vs PE+antibody, p=1; Control+antibody vs PE+IgG, p=0.001; Control+antibody vs PE+antibody, p=0.75; PE+IgG vs PE+antibody, p=0.014;

Panel B: for male offspring, One-way ANOVA, Tukey HSD, F(3,60)=3.112, p=0.033;

Control+IgG vs Control+antibody, p=0.954; Control+IgG vs PE+IgG, p=0.105; Control+IgG vs PE+antibody, p=1; Control+antibody vs PE+IgG, p=0.03; Control+antibody vs PE+antibody, p=0.869; PE+IgG vs PE+antibody, p=0.173;

for female offspring, One-way ANOVA, Tukey HSD, F(3,60)=0.229, p=0.876;

Control+IgG vs Control+antibody, p=0.912; Control+IgG vs PE+IgG, p=1; Control+IgG vs PE+antibody, p=0.949; Control+antibody vs PE+IgG, p=0.926; Control+antibody vs PE+antibody, p=1; PE+IgG vs PE+antibody, p=0.958;

Panel C: for male offspring, One-way ANOVA, Tukey HSD, F(3,76)=1.062, p=0.37;

Control+IgG vs Control+antibody, p=0.885; Control+IgG vs PE+IgG, p=0.759; Control+IgG vs PE+antibody, p=0.965; Control+antibody vs PE+IgG, p=0.323; Control+antibody vs PE+antibody, p=0.628; PE+IgG vs PE+antibody, p=0.956;

for female offspring, One-way ANOVA, Tukey HSD, F(3,80)=0.1.101, p=0.354;

Control+IgG vs Control+antibody, p=0.748; Control+IgG vs PE+IgG, p=1; Control+IgG vs PE+antibody, p=0.829; Control+antibody vs PE+IgG, p=0.768; Control+antibody vs PE+antibody, p=274; PE+IgG vs PE+antibody, p=0.898.

**Fig.S10:**

Panel B:

One-way ANOVA, Tukey HSD; F(7,979)=53.331, p＜0.001;

for male offspring:

Control+IgG vs Control+antibody, p=0.991;

Control+IgG vs PE+IgG, p＜0.001;

Control+IgG vs PE+antibody: p=0.994;

Control+antibody vs PE+IgG, p＜0.001;

Control+antibody vs PE+antibody, p=1.0;

PE+IgG vs PE+antibody, p＜0.001;

for female offspring:

Control+IgG vs Control+antibody, p=0.773;

Control+IgG vs PE+IgG, p＜0.001;

Control+IgG vs PE+antibody: p=0.991;

Control+antibody vs PE+IgG, p＜0.001;

Control+antibody vs PE+antibody, p=0.235;

PE+IgG vs PE+antibody, p＜0.001;

Panel D:

One-way ANOVA, Tukey HSD; F(7,993)=8.587, p＜0.001;

for male offspring:

Control+IgG vs Control+antibody, p=1.0;

Control+IgG vs PE+IgG, p＜0.001;

Control+IgG vs PE+antibody: p=1.0;

Control+antibody vs PE+IgG, p＜0.001;

Control+antibody vs PE+antibody, p=1.0;

PE+IgG vs PE+antibody, p＜0.001;

for female offspring:

Control+IgG vs Control+antibody, p=0.985;

Control+IgG vs PE+IgG, p＜0.001;

Control+IgG vs PE+antibody: p=1.0;

Control+antibody vs PE+IgG, p=0.023;

Control+antibody vs PE+antibody, p=0.235;

PE+IgG vs PE+antibody, p=0.002.

**Fig.S11:**

Panel B:

Two-way ANOVA, Tukey HSD, F(6, 96)=6.33, p＜0.001；

p-S468 Nfκb:

Con+IgG vs Con+antibody, p=0.996;

Con+IgG vs PE+IgG, p=0.003;

Con+IgG vs PE+antibody, p=0.973;

Con+antibody vs PE+IgG, p=0.002;

Con+antibody vs PE+antibody, p=0.997;

PE+IgG vs PE+antibody, p＜0.001.

p-S536 Nfκb:

Con+IgG vs Con+antibody, p=0.999;

Con+IgG vs PE+IgG, p＜0.001;

Con+IgG vs PE+antibody, p=1;

Con+antibody vs PE+IgG, p＜0.001;

Con+antibody vs PE+antibody, p=1;

PE+IgG vs PE+antibody, p＜0.001.

total Nfκb:

Con+IgG vs Con+antibody, p=1;

Con+IgG vs PE+IgG, p=0.996;

Con+IgG vs PE+antibody, p=1;

Con+antibody vs PE+IgG, p=0.98;

Con+antibody vs PE+antibody, p=1;

PE+IgG vs PE+antibody, p=0.983.

Panel C:

Two-way ANOVA, Tukey HSD, F(24, 180)=6.524, p＜0.001;

PD10, PD11, PD12, all comparison, p＞0.05;

PD 13, PD 14, PD 15, PD 16, PD 17, PD 18, PE+IgG vs Control+IgG, all comparison, p＜0.001;

PE+antibody vs Control+IgG, all comparison, p＜0.001，except PD14, p=0.001;

PE+antibody vs Control+antibody, all comparison, p＜0.001.

Panel D, mortality, Kruskal-Wallis with Dunn’s correction test: p=0.001

Control+IgG vs Control+antibody, p＞0.99;

Control+IgG vs PE+IgG, p=0.008;

Control+IgG vs PE+antibody, p=0.029

Control+antibody vs PE+IgG, p=0.015

Control+antibody vs PE+antibody, p=0.046

PE+IgG vs PE+antibody, p＞0.99.

Panel E:

One-way ANOVA, Tukey HSD.

Body weight offspring at E17.5: F(3,28)=37.67, p＜0.001;

Control+IgG vs Control+antibody, p=0.997;

Control+IgG vs PE+IgG, p＜0.001;

Control+IgG vs PE+antibody, p＜0.001;

Control+antibody vs PE+IgG, p＜0.001;

Control+antibody vs PE+antibody, p＜0.001;

PE+IgG vs PE+antibody, p=0.438.

Body weight offspring at P0: F(3,28)=46.67, p＜0.001;

Control+IgG vs Control+antibody, p=0.612;

Control+IgG vs PE+IgG, p＜0.001;

Control+IgG vs PE+antibody, p＜0.001;

Control+antibody vs PE+IgG, p＜0.001;

Control+antibody vs PE+antibody, p＜0.001;

PE+IgG vs PE+antibody, p=0.916.

Panel F:

One-way ANOVA, Tukey HSD,

Brain weight offspring at E17.5: F(3,28)=46.05, p＜0.001;

Control+IgG vs Control+antibody, p=0.989;

Control+IgG vs PE+IgG, p＜0.001;

Control+IgG vs PE+antibody, p＜0.001;

Control+antibody vs PE+IgG, p＜0.001;

Control+antibody vs PE+antibody, p＜0.001;

PE+IgG vs PE+antibody, p=0.891.

Brain weight offspring at P0: F(3,28)=8.826 p＜0.001;

Control+IgG vs Control+antibody, p=0.998;

Control+IgG vs PE+IgG, p=0.008;

Control+IgG vs PE+antibody, p=0.006;

Control+antibody vs PE+IgG, p=0.005;

Control+antibody vs PE+antibody, p=0.004;

PE+IgG vs PE+antibody, p=0.997.

Panel G:

One-way ANOVA, Tukey HSD,

Brain/body weight ratio offspring at E17.5: F(3,28)=11.48, p＜0.001;

Control+IgG vs Control+antibody, p=0.99;

Control+IgG vs PE+IgG, p＜0.001;

Control+IgG vs PE+antibody, p=0.006;

Control+antibody vs PE+IgG, p＜0.001;

Control+antibody vs PE+antibody, p=0.012;

PE+IgG vs PE+antibody, p=0.665.

Brain/body weight ratio offspring at P0: F(3,28)=14.98, p＜0.001;

Control+IgG vs Control+antibody, p=0.706;

Control+IgG vs PE+IgG, p=0.002;

Control+IgG vs PE+antibody, p=0.002;

Control+antibody vs PE+IgG, p＜0.001;

Control+antibody vs PE+antibody, p＜0.001;

PE+IgG vs PE+antibody, p＞0.9.

Panel H:

Two-way ANOVA, Tukey HSD, Body weight offspring.

Control +IgG vs PE+IgG, P0, p＜0.001; P2, p＜0.001; P3, p＜0.001; other time points, all p＞0.05.

Control+antibody vs PE+antibody, P0, p＜0.001; P2, p＜0.001; P3, p＜0.001; other time points, all p＞0.05.

Control+IgG vs Control antibody, all p＞0.05.

PE+IgG vs PE+antibody, all p＞0.05.

Panel I:

Two-way ANOVA, Tukey HSD, F(24, 180)=0.471, p=0.982; water consumption per day. All comparison, p＞0.05.

References:

1. Li Q, Verma A, Han PY, Nakagawa T, Johnson RJ, Grant MB, Campbell-Thompson M, Jarajapu YP, Lei B, Hauswirth WW (2010) Diabetic enos-knockout mice develop accelerated retinopathy. *Investigative ophthalmology & visual science* 51: 5240-5246. doi:10.1167/iovs.09-5147

2. Bachmanov AA, Reed DR, Beauchamp GK, Tordoff MG (2002) Food intake, water intake, and drinking spout side preference of 28 mouse strains. *Behav Genet* 32: 435-443. doi:10.1023/a:1020884312053

3. Choi GB, Yim YS, Wong H, Kim S, Kim H, Kim SV, Hoeffer CA, Littman DR, Huh JR (2016) The maternal interleukin-17a pathway in mice promotes autism-like phenotypes in offspring. *Science* 351: 933-939. doi:10.1126/science.aad0314

4. Liu X, Zhao W, Liu H, Kang Y, Ye C, Gu W, Hu R, Li X (2016) Developmental and functional brain impairment in offspring from preeclampsia-like rats. *Mol Neurobiol* 53: 1009-1019. doi:10.1007/s12035-014-9060-7

5. de Fiebre NC, Sumien N, Forster MJ, de Fiebre CM (2006) Spatial learning and psychomotor performance of c57bl/6 mice: Age sensitivity and reliability of individual differences. *Age (Dordrecht, Netherlands)* 28: 235-253. doi:10.1007/s11357-006-9027-3
